# Enhancing Underwater SLAM Navigation and Perception: A Comprehensive Review of Deep Learning Integration

**DOI:** 10.3390/s24217034

**Published:** 2024-10-31

**Authors:** Fomekong Fomekong Rachel Merveille, Baozhu Jia, Zhizun Xu, Bissih Fred

**Affiliations:** 1 School of Naval Architecture and Maritime, Guangdong Ocean University, Zhanjiang 524000, China; marvelous@stu.gdou.edu.cn; 2 School of Engineering, Newcastle University, Newcastle upon Tyne NE1 7RU, UK; zhizun@gdou.edu.cn; 3 College of Fisheries, Guangdong Ocean University, Zhanjiang 524088, China; 1252201246@stu.gdou.edu.cn

**Keywords:** underwater simultaneous localization and mapping (SLAM), underwater navigation, deep learning, odometry navigation

## Abstract

Underwater simultaneous localization and mapping (SLAM) is essential for effectively navigating and mapping underwater environments; however, traditional SLAM systems have limitations due to restricted vision and the constantly changing conditions of the underwater environment. This study thoroughly examined the underwater SLAM technology, particularly emphasizing the incorporation of deep learning methods to improve performance. We analyzed the advancements made in underwater SLAM algorithms. We explored the principles behind SLAM and deep learning techniques, examining how these methods tackle the specific difficulties encountered in underwater environments. The main contributions of this work are a thorough assessment of the research into the use of deep learning in underwater image processing and perception and a comparison study of standard and deep learning-based SLAM systems. This paper emphasizes specific deep learning techniques, including generative adversarial networks (GANs), convolutional neural networks (CNNs), long short-term memory (LSTM) networks, and other advanced methods to enhance feature extraction, data fusion, scene understanding, etc. This study highlights the potential of deep learning in overcoming the constraints of traditional underwater SLAM methods, providing fresh opportunities for exploration and industrial use.

## 1. Introduction

The Earth’s oceans, which span over 71% of the planet’s surface, are precious resources for scientific investigation and environmental understanding [1,2]. Unmanned underwater vehicles (UUVs) [3,4] have a variety of crucial applications, including marine mining and pipeline inspection [5,6,7]. However, their efficacy is frequently hampered by the limits of conventional navigation systems [8,9]. Inertial sensors and acoustic beacons used for underwater navigation suffer from accumulated errors, limited range, and environmental interference [9,10]. Furthermore, optical problems such as low lighting, turbidity, scattering, and wavelength absorption affect visual dependability [11]. The lack of access to global positioning systems (G.P.S.) [12,13,14] affects accurate location determination and data collecting in underwater situations.

The difficulties of underwater navigation have increased the demand for more dependable and precise solutions. Recent advances in deep learning [15], particularly in visual simultaneous localization and mapping (SLAM) [16], have revealed intriguing areas for improvement. Significant progress has been achieved in tackling the specific constraints of underwater environments, resulting in enhanced capabilities for unmanned underwater vehicles (UUVs) [17,18]. This study investigates these improvements, evaluating their potential to transform underwater navigation and perception.

This paper reviews crucial studies on how deep learning improves underwater SLAM systems. The literature selection methodology includes comprehensive searches in scientific databases such as IEEE Xplore, SpringerLink, ScienceDirect, Nature, and Google Scholar using keywords such as “underwater SLAM”, “deep learning”, “UUV navigation”, “N.N. underwater” [19,20,21,22,23,24,25], “RNN SLAM”, “GAN underwater” [26,27,28], and “V.A.E. mapping”. Papers were selected based on their ability to advance the field, their citation count, their publication in reputable journals and conferences, and their relevance to the incorporation of deep learning techniques [29] into underwater SLAM. Recent developments and seminal works were assessed. To preserve the quality and relevance of the review, papers that were not peer-reviewed, lacked experimental validation, or did not explicitly address underwater applications were excluded.

The chosen articles were classified into different groups according to their main emphasis, including conventional primary techniques, CNNs for extracting features, R.N.N.s for modeling temporal aspects, DRL for modeling and mapping, GANs [30] for augmenting data, and V.A.E.s for exploring latent space. Every publication underwent a thorough assessment to identify and extract the main contributions, methodology or processes, results, and implications for the discipline. To enhance the understanding of the status of the field, we emphasized the most influential and significant papers in each area, ensuring a well-organized and comprehensive overview. This study seeks to give a clear roadmap for academics and practitioners interested in advancing the area of underwater simultaneous localization and mapping (SLAM) by integrating deep learning techniques [15,31]. It accomplishes this by categorizing the existing literature and emphasizing the most influential studies.

There are eight main sections in this paper: (1)Introduction, which establishes the study goals and relevance, gives background information on underwater SLAMs, and emphasizes the function of deep learning; (2) progress in algorithmic underwater SLAM with an emphasis on performance in underwater environments, which objectively assesses the most recent advancements in SLAM technologies; (3) mathematical foundations of underwater SLAM and deep learning approaches, which presents the theoretical models underlying these methods; (4) deep learning uses in underwater perception, navigation, and image processing, which examines deep learning techniques for enhancing underwater sensing and navigation; (5) strengths and limitations of deep learning-based underwater SLAM and odometry navigation, which provides a review of the benefits and drawbacks of deep learning for SLAM; (6) comparative analysis of underwater SLAM techniques, which evaluates the resilience and efficiency of several SLAM strategies; (7) deep learning’s superiority over conventional methods, which emphasizes the technology’s benefits in solving undersea problems; and (8) conclusion and following directions, which emphasizes deep learning’s developing importance in underwater SLAM, summarizes findings, and recommends following research directions.

## 2. Common Underwater SLAM Advancements and Algorithm Performance

SLAM has advanced, especially with the extended Kalman filter (E.K.F.) [32] SLAM for probabilistic robot pose and landmark estimation. Due to its linearization assumptions, EKF SLAM [33] struggles in complicated nonlinear situations despite its popularity. The square root information filter (SRIF) [34] algorithm addresses these issues. By controlling the covariance matrix and resolving numerical instability, SRIF improves underwater UUV navigation stability, numerical reliability, and operational efficiency.

Different SLAM methods address various issues in various contexts. Particle filter SLAM [35] thrives in highly nonlinear settings, while graph-based SLAM [36] optimizes graph representations for configuration detection. Bayesian and Rao–Blackwellized particle filter (RBPF) SLAMs increase mapping and localization using Bayesian [37] estimation and computational efficiency, respectively.

Underwater sensor constraints must be addressed. Innovative technologies like sonar-based [38] mapping and loop closure detection improve SLAM performance.

Visual SLAM [39] algorithms for underwater use have been tested. ORB-SLAM [40] performs best in well-lit and feature-rich situations, while ROVIO [41] uses visual and inertial data to excel in dynamic underwater environments. LSD-SLAM [42] maps perform well in texture-rich underwater environments, and DVO-SLAM [43,44] handles depth changes well. The multi-state constraint Kalman filter (MSCKF) [45,46] uses visual and inertial measurements for precise navigation, and SVO [47,48] is efficient for lightweight underwater vehicles. Deep learning-enhanced [49] VISLAM [50] algorithms can extract and map features in challenging underwater settings. FAB-MAP [51], designed for underwater applications, performs well in varied underwater environments with distinct visual aspects.

**Figure 1:**
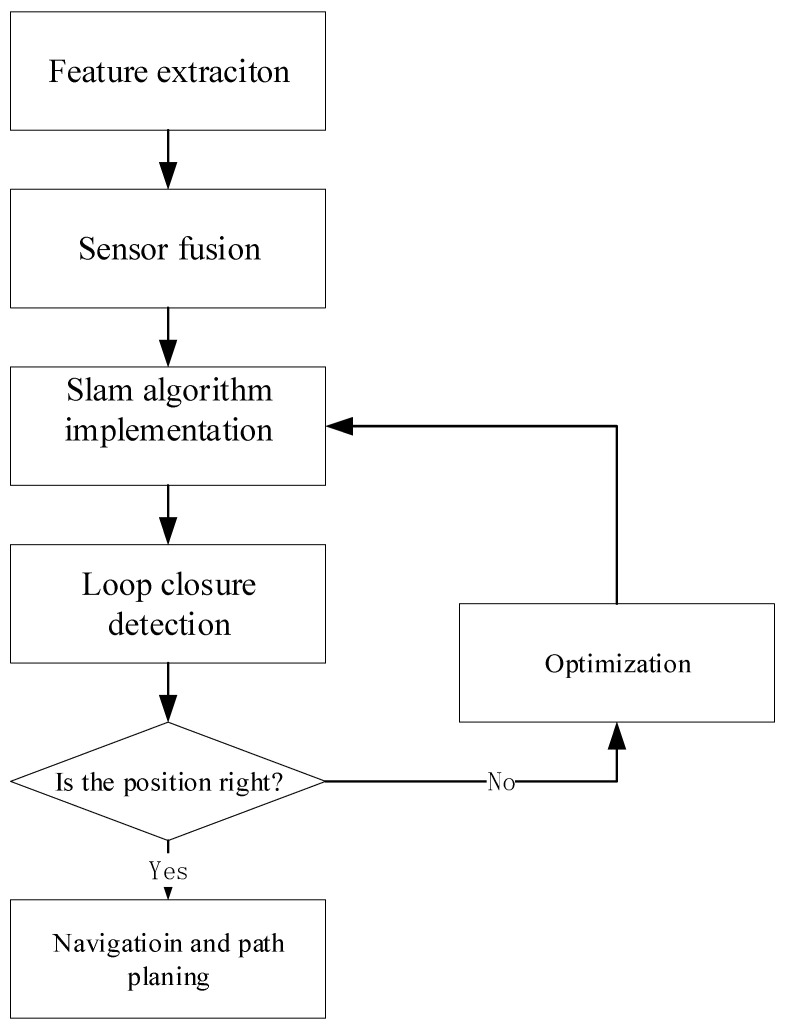
Underwater SLAM process.

Recent advances in underwater SLAM systems, as shown in Figure 1, have improved navigation accuracy. Even though underwater navigation is complex, deep learning and SLAM have improved UUVs for oceanographic investigation [52]. Deep learning improves SLAM by employing neural networks to enhance mapping and navigation, especially with LiDAR and vision sensors [53,54]. Regularly distributed artificial magnetic beacons can provide a repeated regional magnetic field, allowing autonomous cars to navigate with high positioning accuracy by considering landmarks for SLAM algorithms [55]. The VINS-MONO [56] technique uses FAST feature point extraction and inverse optical flow to increase speed and accuracy, addressing underwater picture degradation [57]. End-to-end networks for low-light SLAM preprocessing have also been developed with a low-light improvement branch and a self-supervised feature point detector to increase feature point extraction and reduce re-projection mistakes [58].

Generative adversarial networks (GANs) [27] for real-time underwater image improvement have improved SLAM performance by tackling poor visibility, low contrast, and color distortion, providing robust and accurate monocular SLAM systems. In unknown underwater environments, multi-vehicle collaborative mapping is more efficient due to algorithms like Gaussian mixture robust branch and bound (GMRBnB) that improve map registration accuracy and outlier tolerance [59]. Improved unscented Kalman filter SLAM (IUKF-SLAM) was designed to improve the accuracy, consistency, and convergence of the unscented Kalman filter (U.K.F.) [60] used in SLAM, displaying a better performance compared to existing E.K.F. [33,61] and U.K.F. approaches [62]. Underwater SLAM combines several information sources to overcome the limits of standard navigation systems, particularly in complex and unstructured underwater landscapes, allowing high-precision navigation and placement even without satellite information [63].

These advances in underwater SLAM algorithms, including deep learning integration, magnetic beacon use, image enhancement, collaborative mapping, and improved filtering, have greatly enhanced underwater navigation systems’ accuracy and reliability, making systems more autonomous and effective in underwater exploration and operations.

## 3. Mathematical Formulas for Underwater SLAM and Deep Learning Methods

Underwater SLAM uses a variety of mathematical formulas and algorithms to map and navigate effectively. The PF-backend (particle filter-backend) approach utilizes particle filters for loop closure estimates and map consistency [64]. Variational Bayesian (V.B.) learning estimates UUV route and observation noise using the inverse-gamma distribution. The Gaussian mixture robust branch and bound (GMRBnB) technique improves map registration by extracting features and inliers. For robust SLAM, dual-stage bathymetric data association and Euler-deconvolution algorithms localize magnetic beacons. For real-time location, coalition game theory optimizes artificial magnetic beacon distribution. Neural network-detected semantic landmarks are used in object-level SLAM [65]. The preparation of mechanically scanned imaging sonar (MSIS) data allows for the utilization of traditional 2D laser SLAM frameworks, such as Gmapping and Cartographer, to tackle obstacles encountered underwater [66,67]. These methods jointly enhance the accuracy and dependability of SLAM by tackling problems such as error accumulation and feature extraction in low-texture situations. Below are some derivations of the mathematical formulae used in SLAM.

E.K.F. State Update
  (1) $$X_{k|k}=X_{k|k-1}+K_{k}(Z_{k}-h(X_{k|k-1}))$$

$X_{k|k}$: Update state estimate, $Z_{k}$: Measurement at time *k*, h: Measurement function.

E.K.F. Covariance Update
  (2) $$P_{k|k}=\;\left(I-K_{k}H_{k}\right)P_{k|k-1}$$

$P_{k|k}$: Update covariance estimate, I: Identity matrix.

Particle Filter Weight Update
  (3) $$W_{t}\left[i\right]\;\infty \;W_{t-1}\left[i\right].p\left(z_{t}|\;x_{t}\left[i\right],\;m_{t-1}\right)$$

$W_{t}\left[i\right]$: Weight of the particle *i* at time *t*, $z_{t}$: Measurement at time *t*,

$x_{t}\left[i\right]$: State of particle i at time t, $m_{t-1}$: Map at time t − 1.

Particle Filter Resampling
  (4) $$\left\{X_{t}\left[i\right]\right.{,W_{t}\left.\left[i\right]\right\}}_{i=1}^{N}\;\sim \left\{X_{t}\left[i\right]\right.{,{\tilde{W}}_{t}\left.\left[i\right]\right\}}_{i=1}^{N}$$

$X_{t}\left[i\right]$: State of particle i, $W_{t}\left[i\right]$: Weight of particle i, ${\tilde{W}}_{t}\left[i\right]$: Normalized weight of the particle i.

Convolution Operation
  (5) $$\left(I*K\right)\left(i,\;j\right)=\sum_{m}\sum_{n}I\left(i+m,\;j+n\right).\;K(m,n)\;$$

I: Input image, K: Kernel, (i, j): Position in the output image.

ReLU Activation Function
  (6) $$f\left(x\right)=max\left(0,\;x\right)$$

$f\left(x\right)$: Output, x: Input.

R.N.N. Hidden State Update
  (7) $$h_{t}=\sigma_{h}\left(W_{h}x_{t}+U_{h}h_{t-1}+b_{n}\right)$$

$h_{t}$: Hidden state at time t, $\sigma_{h}$: Activation function, $W_{h}$: Weight matrix for input, $x_{t}$: Input at time t, $U_{h}$: Weight matrix for the hidden state, $b_{n}$: Bias term.

LSTM Input Gate
  (8) $$i_{t}=\sigma (W_{i}x_{t}+U_{i}h_{t-1}+b_{i})$$

$i_{t}$: Input gate at time t, σ: Sigmoid function, $W_{i}$: Weight matrix for input, $x_{t}$: Input at time t, $U_{i}$: Weight matrix for the hidden state, $b_{i}$: Bias term.

LSTM Output Gate
  (9) $$O_{t}=\sigma (W_{0}x_{t}+U_{0}h_{t-1}+b_{0})$$

$O_{t}$: Output gate at time t, σ: Sigmoid function, $W_{0}$: Weight matrix for input, $x_{t}$: Input at time t, $U_{0}$: Weight matrix for the hidden state, $b_{0}$: Bias term.

LSTM Hidden State Update
  (10) $$h_{t}=O_{t}⊙tanh(c_{t})$$

$h_{t}$: Hidden state at time t, $O_{t}$: Output gate at time t, tanh: Hyperbolic tangent function, $c_{t}$: Cell state at time t.

Bellman Equation
  (11) $$Q\left(s,a\right)=r+\gamma \underset{a^{\prime }}{max}Q\left(s^{\prime },a^{\prime }\right)$$

$Q\left(s,a\right)$: State-action value, r: Reward, γ: Discount factor, $a^{\prime }$: Next action, $s^{\prime }$: Next state.

GAN Loss Functions
  (12) $$\underset{G}{min}\underset{D}{max}V\left(D,G\right)=E_{x\sim p_{data}\left(x\right)}\left[logD\left(x\right)\right]+E_{z\sim p_{z}\left(z\right)}\left[log\left(1-D\left(G\left(Z\right)\right)\right)\right]$$

G: Generator, D: Discriminator, x: Real data, z: Latent variable, $p_{data}(x)$: Data distribution, $p_{z}\left(z\right)$: Latent distribution.

V.A.E. Loss Function
  (13) $$(L\left(x,z\right)=E_{q_{\phi }\left(z|x\right)}[logp_{\theta }\left(x|z\right)-D_{KL}\left(q_{\theta }\left(z|x\right)∥p_{\theta }\left(z\right)\right)$$

L(x, z): VAE loss, $q_{\theta }\left(z|x\right)$: Approximate posterior, $p_{\theta }\left(x|z\right)$: Likehood, $D_{KL}$: Kullback-Leibler divergence.

In SLAM, state estimation is vital for accuracy and efficiency. The E.K.F. handles nonlinear systems, predicting and updating states using sensor data. Equations (1) and (2) guide state updating and covariance updates, and Kalman gain could also be added to minimize uncertainty. For more complex non-Gaussian environments, particle filters (Equations (3) and (4)) offer robust alternatives by assigning particle weights and resampling for diversity.

Deep learning models enhance feature extraction in SLAM through convolutions (Equation (5)) and ReLU activation (Equation (6)). Recurrent neural networks (R.N.N.s) and LSTMs (Equations (7)–(10)) handle temporal dependencies for more stable mapping in dynamic environments. Reinforcement learning (Equation (11)) optimizes exploration, while GANs and V.A.E.s (Equations (12) and (13)) generate environmental models, improving SLAM’s robustness in unstructured spaces.

## 4. Deep Learning Techniques in Underwater Image Processing, Navigation, and Perception

Deep learning techniques are a groundbreaking approach in machine learning that involves using neural networks with numerous layers to analyze and understand intricate patterns in data. Convolutional neural networks (CNNs) [68] are highly effective in image tasks, as they can capture hierarchical features for tasks like image recognition and computer vision applications. On the other hand, recurrent neural networks (R.N.N.s), play a crucial role in processing sequential data, such as language and time-series information, because they can retain context and dependencies. Transfer learning strategies utilize pre-trained models to enhance performance on specific tasks, enabling efficient information transfer. Generative adversarial networks (GANs) [27,28,29,30] introduce a new method for generating realistic data. Advanced natural language processing (N.L.P.) models, such as BERT and G.P.T., demonstrate exceptional proficiency in comprehending and producing language that resembles human speech. Deep learning techniques [29,69] have been widely used in underwater applications to improve the accuracy of visual perception systems. These techniques have been applied in several areas, such as image processing, navigation, and perception, and have shown impressive proficiency in object detection, recognition, and segmentation tasks. Transfer learning has successfully addressed the problem of limited data availability, leading to encouraging outcomes in different undersea situations. In addition, integrating sensor data, such as sound and visual inputs, using deep learning architectures improves perceptual abilities, resulting in more precise mapping and better comprehension of the surroundings.

**Figure 2:**
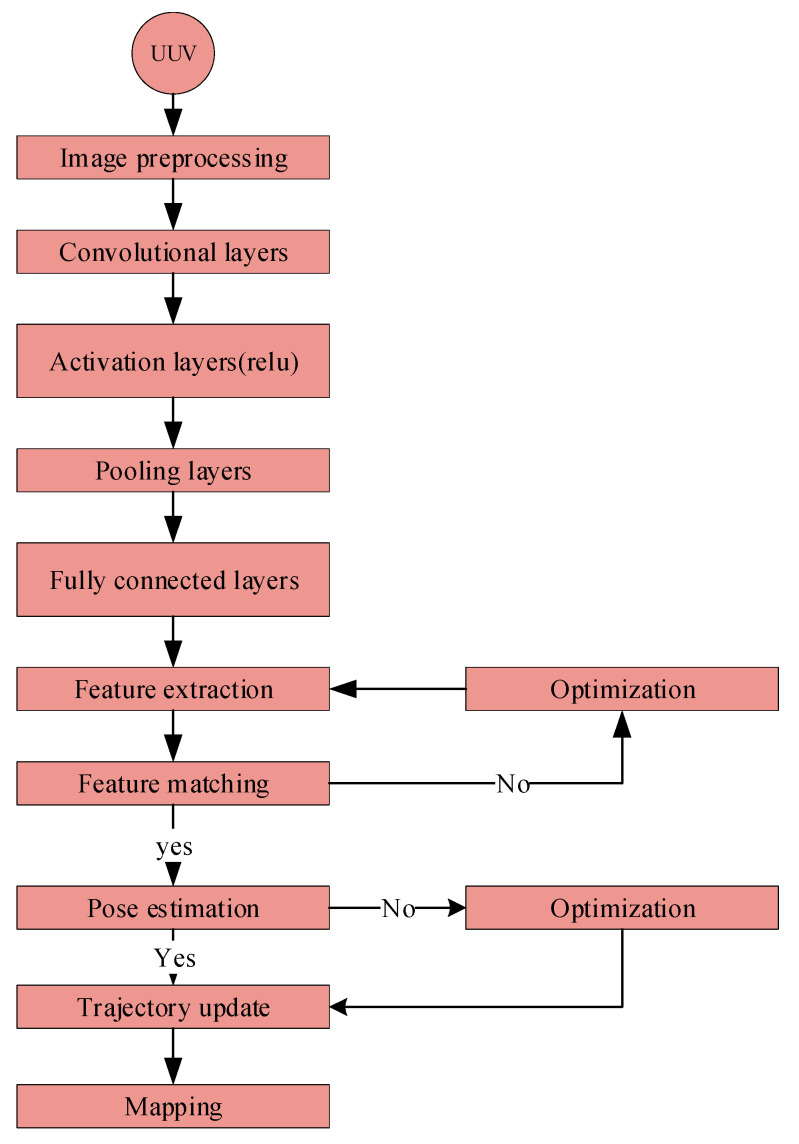
Deep CNNs in underwater SLAM process. This flow chart shows how deep CNNs [70] are used for underwater SLAMs. Images are captured using a UUV camera and preprocessed for visibility. For feature extraction, preprocessed pictures are routed via convolutional, activation, and pooling layers. Fully connected layers provide a high-level visual knowledge and match features between frames. Matching features estimate pose, update the robot’s trajectory, and map the environment.

Deep learning has dramatically improved underwater image processing, navigation, and perception, effectively tackling specific issues. Methods such as VDSR and BCFO-based deep CNN enhance the clarity of images and object detection accuracy [21,70]. The FUnIE-GAN architecture enhances visual perception measures such as PSNR (peak signal-to-noise ratio), SSIM (structural similarity index), UCIQE (universal image quality index), and entropy [71]. The combination of VGG16 and visual saliency models improves image clarity and enhances the accuracy of colors [72]. Deep learning enhances underwater perception by using advanced techniques such as RCNN (region convolutional neural network) and CFTA (color filter tensor analysis). These techniques specifically target the challenges of inadequate illumination and limited visibility in underwater environments [73]. Surveys indicate that deep learning performs more remarkably in effectively managing difficulties like underwater turbulence, low contrast, and color distortion [74]. Convolutional neural networks (CNNs) illustrated in Figure 2, are highly proficient in the classification and detection of underwater species and objects, hence assisting in the protection of marine ecosystems [19]. These improvements enable new opportunities for exploring the ocean as well as different industrial uses (Table 1 and Table 2 elaborate on the comparative analysis of the traditional deep learning-based underwater SLAM techniques).

**Figure 3:**
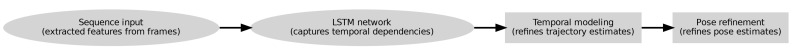
Underwater SLAM RNN process.

The underwater SLAM RNN process using long short-term memory (LSTM) and recurrent neural networks (R.N.N.s) for underwater SLAM are shown in Figure 3. A sequence of retrieved features from consecutive frames is sent into the LSTM network to capture temporal dependencies. Temporal modeling via the LSTM network refines the robot’s trajectory. Finally, the LSTM output refines feature-matching pose predictions for accurate and efficient underwater SLAM. 

**Figure 4:**
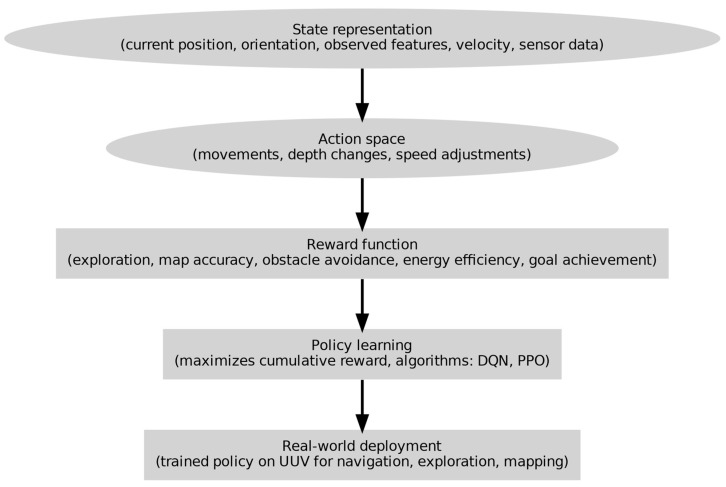
Detailed procedure for underwater SLAM for UUV using deep reinforcement learning (DRL).

The procedures involved in employing DRL for underwater SLAM are shown in Figure 4. The state representation, which includes the current position, orientation, velocity, observed features, and sensor data, is where the process begins. The action space delineates the range of conceivable motions, depth modifications, and velocity changes. The reward function promotes goal achievement, energy efficiency, obstacle avoidance, map accuracy, and exploration. Algorithms such as D.Q.N. and P.P.O. are used in policy learning to maximize cumulative rewards. Lastly, a UUV equipped with the trained policy is used for mapping, exploration, and navigation. 

**Figure 5:**
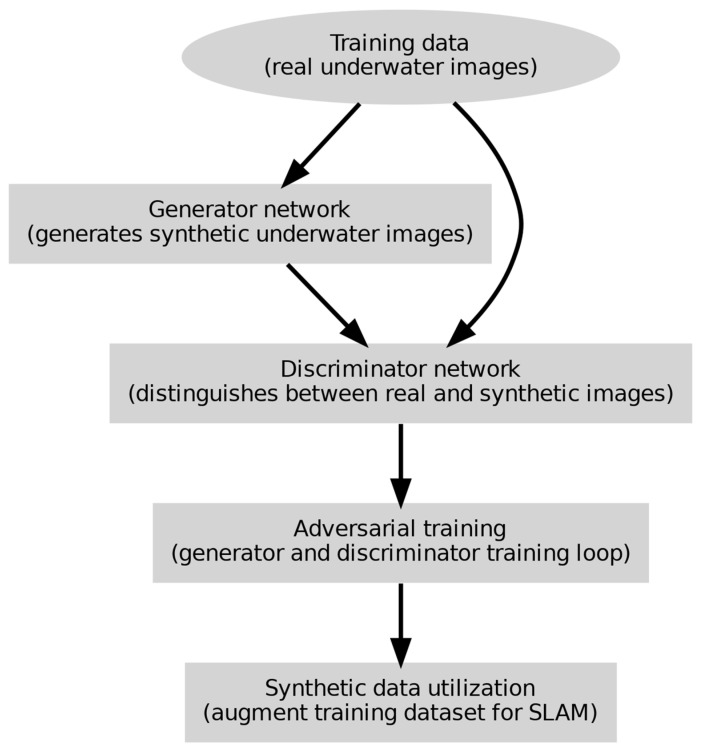
Detailed GAN process in underwater SLAM for UUV.

Figure 5 shows how GANs generate realistic underwater images for SLAM deep learning model training. Real underwater photos are used to train the GAN. The discriminator network separates real from manufactured underwater images while the generator generates them. Adversarial training loops the generator and discriminator to increase visual realism. Synthetic data is added to the training dataset for SLAM jobs to improve underwater SLAM algorithm performance and robustness.

**Figure 6:**
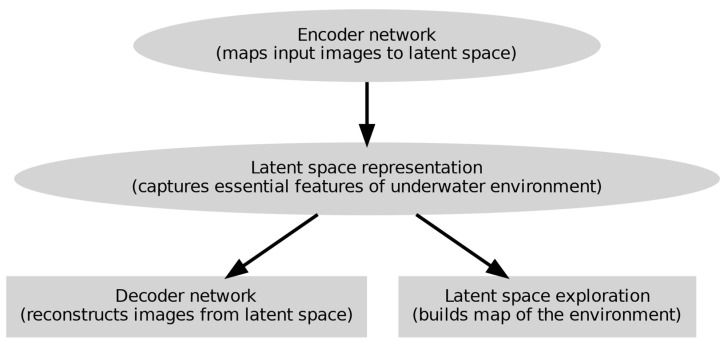
Comprehensive variational autoencoders.

Figure 6 shows a detailed approach of V.A.E.s in underwater SLAM for UUVs.

The procedures for applying V.A.E.s for unsupervised learning of latent representations of underwater environments are shown in Figure 6. Starting with the encoder network, which maps input images to a latent space capturing the salient characteristics of the underwater environment, the process moves from the latent space and the decoder network then rebuilds the photos. Then, a map of the surroundings is created using the latent space representations, enabling efficient underwater SLAM. 

**Figure 7:**
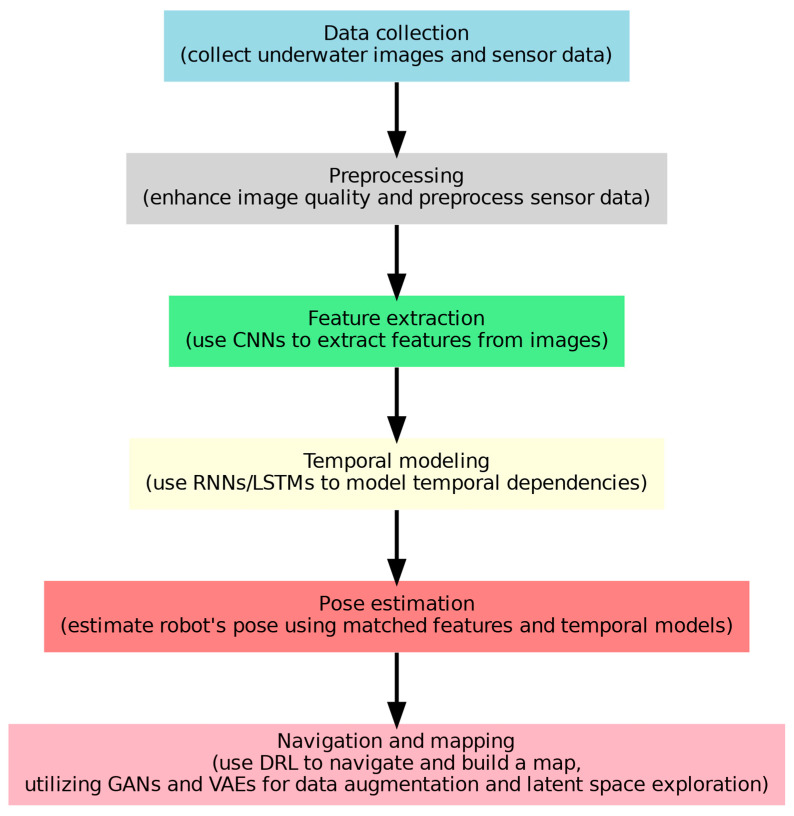
Workflow integration for UUV underwater SLAM.

Figure 7 aggregates the whole underwater SLAM in the UUVs process. Data collecting starts the process; underwater photos and sensor data are obtained. Image quality is improved, and sensor data is preprocessed in the preprocessing phase. CNNs [75] are used in feature extraction to extract important picture features. Recurrent neural networks (R.N.N.s) or long short-term memory (LSTM) networks represent temporal dependencies in temporal modeling. Pose estimation computes the robot’s posture with temporal modeling features. With generative adversarial networks (GANs) [27] employed for data augmentation and latent space exploration, deep reinforcement learning (DRL), shown in, helps navigate and generate a map, facilitating navigation and mapping.

Underwater SLAM systems have performed much better when advanced neural network designs like CNNs, GANs, and LSTMs were incorporated to handle issues like low visibility and changing conditions. Teixeira et al. found that deep learning models outperform classical approaches in underwater settings, improving visual odometry accuracy [76]. CNNs are particularly good at extracting features from underwater data, which makes them useful for tasks like multi-target tracking and habitat mapping [77,78,79]. Neural networks improve AUV (Autonomous underwater vehicle) navigation and obstacle avoidance, enabling them to adapt to changes in their surroundings and system degradation [80,81].

To enable real-time navigation by processing sequential input, LSTM networks process dynamic planning [82,83]. Also, deep networks make possible model-free localization, efficiently managing noise and variability [84,85]. According to some researchers, unsupervised neural networks increase mapping efficiency by improving loop detection. However, problems still exist, such as the requirement for massive databases and processing power. The DM-GAN model, which improves depth map accuracy, is an example of how GANs support dense mapping in monocular SLAM [86]. Furthermore, GANs, aid in path planning by producing tenable routes for self-navigating systems [87].

Adding deep learning to SLAM increases accuracy by enhancing feature extraction and loop closure detection [88,89]. Spatial maps are improved by semantic mapping using CNNs. To demonstrate the efficacy of generative models in SLAM tasks, systems such as GEN-SLAM utilize generative modeling for monocular localization [90]. Even with the progress made, there are still difficulties in smoothly combining these models with conventional SLAM frameworks [91] and showing the improvements of deep learning methods in underwater SLAM. The next part of our dissertation elaborates on the strengths and weaknesses of deep learning UUV SLAM (with the workflow illustrated in Figure 7) and visual odometry navigation. 

## 5. Deep Learning-Based Underwater SLAM and Odometry Navigation: Strengths and Weaknesses

Deep learning-based SLAM and odometry [92,93] navigation systems exhibit several strengths. These include advanced feature extraction, where deep learning models identify intricate patterns from sensor data, thus enhancing mapping accuracy and navigation, and the use of end-to-end learning, simplifying navigation and boosting efficiency [3]. Additionally, deep learning [94,95] models can seamlessly integrate information from various sensors, handling diverse data sources coherently. They are capable of modeling nonlinear relationships and adapting to dynamic changes, making them suitable for unpredictable underwater scenarios [24,96,97].

Moreover, transfer learning allows pre-trained models to be adapted for underwater navigation, accelerating the training process and enabling quicker deployment in real-world scenarios [25]. However, these systems face challenges such as high computational demands and the ‘black box’ nature of neural networks, which may raise interpretability concerns [38,98]. Despite these challenges, the advantages presented by deep learning methods, make it a transformative force in advancing underwater SLAM navigation, offering promising avenues for further research and development.

**Figure 15:**
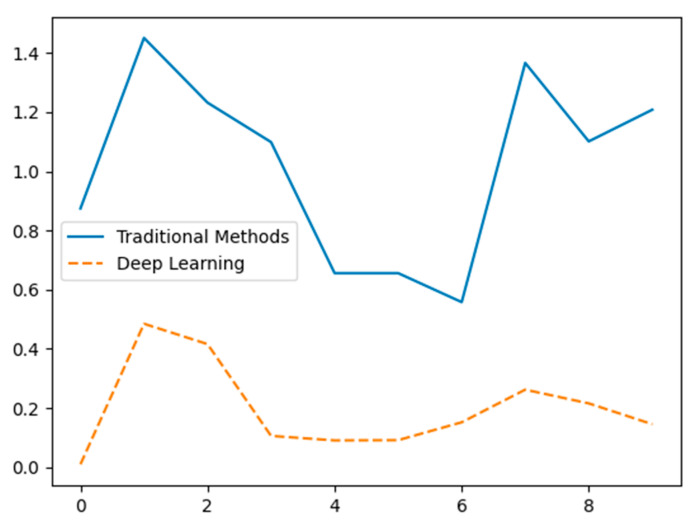
Comparison of real-time processing performance between traditional methods and deep learning using hypothetical data.

**Figure 16:**
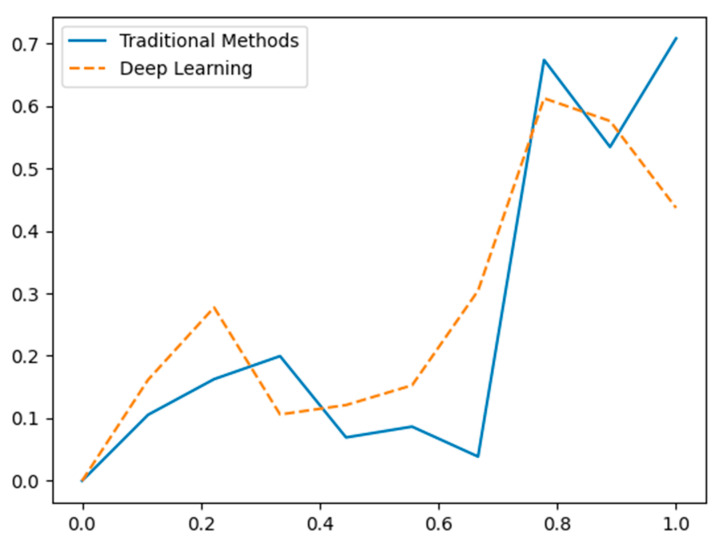
Comparison of the performance in high-level scene understanding between traditional methods and deep learning using hypothetical data.

Underwater SLAM and odometry [93,99,100,101] navigation systems that utilize deep learning have notable benefits, as shown in Figure 15 and Figure 16, but they also encounter certain constraints [102]. These systems demonstrate exceptional performance in intricate and disorganized contexts where conventional approaches frequently prove ineffective. Deep neural networks (N.N.s) improve the precision of underwater acoustic localization (U.A.L.) in environments with reverberation [103]. Generative adversarial networks (GANs) enhance monocular visual SLAM by mitigating issues related to limited visibility and color aberration [26]. Systems like SVIn2, which combine sonar, visual, inertial, and water-pressure data, offer strong performance and dependable initialization [104]. Models with low computational requirements, such as TinyOdom, allow its immediate implementation on devices with few resources [105]. Nevertheless, the effectiveness of these systems relies on acquiring high-caliber training data, a challenging task in underwater environments, impacting accuracy [106]. Additionally, they need substantial processing resources, which pose difficulties for real-time applications on constrained systems. Integrating many sensors becomes more expensive and consumes more power as the complexity increases [107]. Additionally, the performance of neural inertial odometry frameworks might be negatively affected by environmental fluctuations and disturbances [108]. Notwithstanding these difficulties, progress in deep learning and sensor fusion is enhancing UUVs and applications of underwater robotics [109,110,111].

**Figure 8:**
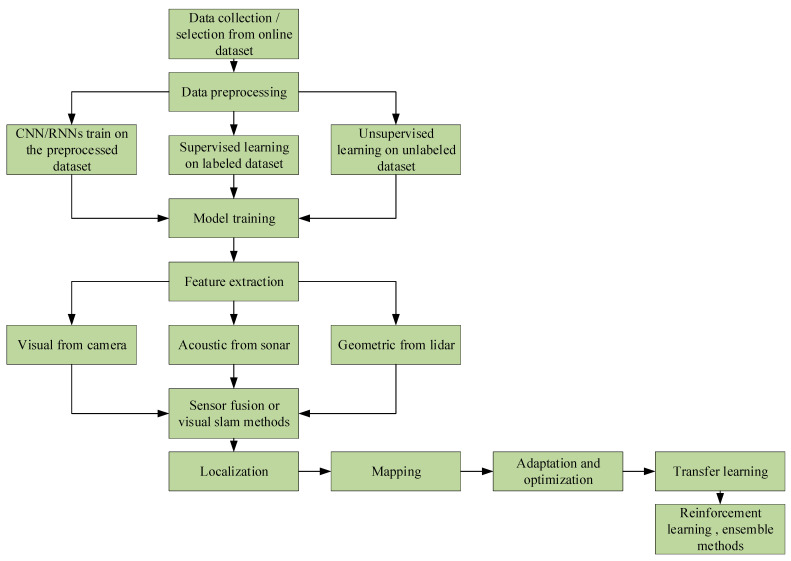
A flowchart depicting the machine learning pipeline for autonomous systems.

Figure 8 illustrates the sequential process from gathering data to implementing learning techniques, including supervised and unsupervised methods. The main steps involved in this process include data preprocessing, model training using CNN/RNN [20,21,23,24,25] architectures, and integrating input from visual, auditory, and geometric sensors in different ways. The process ends with the implementation of localization and mapping techniques, the utilization of optimization strategies, and the use of advanced machine learning techniques such as reinforcement learning and ensemble approaches. Figure 8 offers a thorough outline of the standard procedures used to develop autonomous robotic systems.

**Figure 9:**
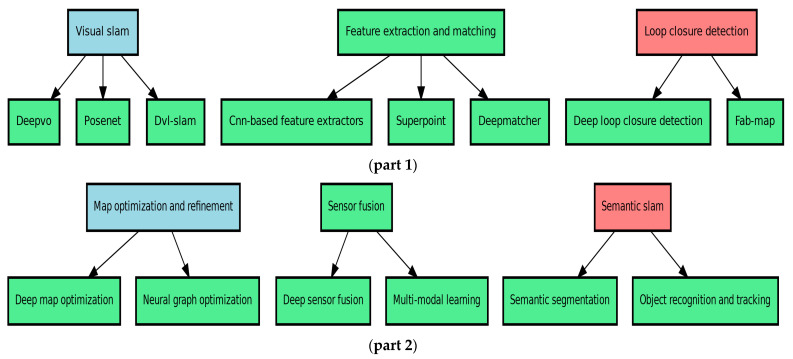
Introduction to deep learning methods for underwater simultaneous localization and mapping (SLAM) (**part 1**, **part 2**).

The diagram in part 1 of Figure 9 depicts the main techniques used in underwater simultaneous localization and mapping (SLAM) using deep learning. These techniques can be classified into the following three basic categories: (1) Visual SLAM, which refers to a collection of methods used for tasks such as visual odometry (DeepVO) [112], pose estimation (PoseNet) [113], and integrating Doppler velocity log data with visual SLAM (DVL-SLAM); (2) feature extraction and matching, which involves methods like CNN-based feature extractors, self-supervised [69] interest point detection and description (SuperPoint) [114], and feature matching in images (DeepMatcher); and (3) loop closure detection, which includes deep loop closure detection using deep learning [115] and probabilistic place recognition based on appearance (FAB-MAP). These technologies jointly improve the precision and durability of underwater SLAM systems, enabling better navigation and mapping in intricate underwater settings.

Figure 9 part 2 depicts the supplemental deep learning approaches utilized in underwater simultaneous localization and mapping (SLAM), categorized into the following three key domains: (1) Map optimization and refinement, which uses sophisticated methods like deep map optimization and neural graph optimization to enhance and streamline underwater maps. Sensor fusion combines data from various sensors, such as deep sensor fusion [116] and multi-modal learning, to improve the performance of SLAM (simultaneous localization and mapping). Semantic SLAM employs techniques like semantic segmentation to comprehend underwater scenes and object recognition and tracking to detect and monitor things in the surroundings. These strategies are crucial for improving underwater SLAM systems’ accuracy, durability, and efficiency.

**Figure 10:**
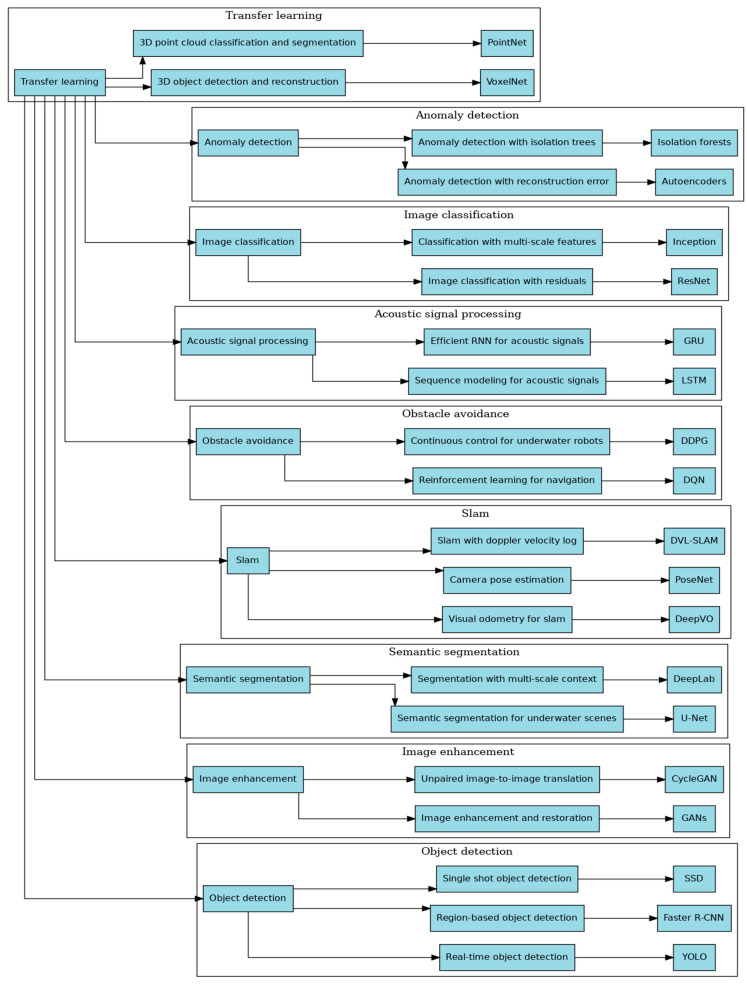
Utilizing transfer learning to leverage deep learning methods [75] for underwater applications.

Figure 10 demonstrates the crucial role of transfer learning [117] in various deep learning techniques used in underwater environments. Transfer learning uses pre-trained models that are subsequently adjusted for specific underwater tasks, including 3D reconstruction, anomaly detection, image classification, acoustic signal processing, obstacle avoidance, SLAM (simultaneous localization and mapping), semantic segmentation, image enhancement, and object detection. Every category uses transfer learning to modify general models for the specific difficulties of undersea applications, ultimately improving performance and efficiency.

**Figure 11:**
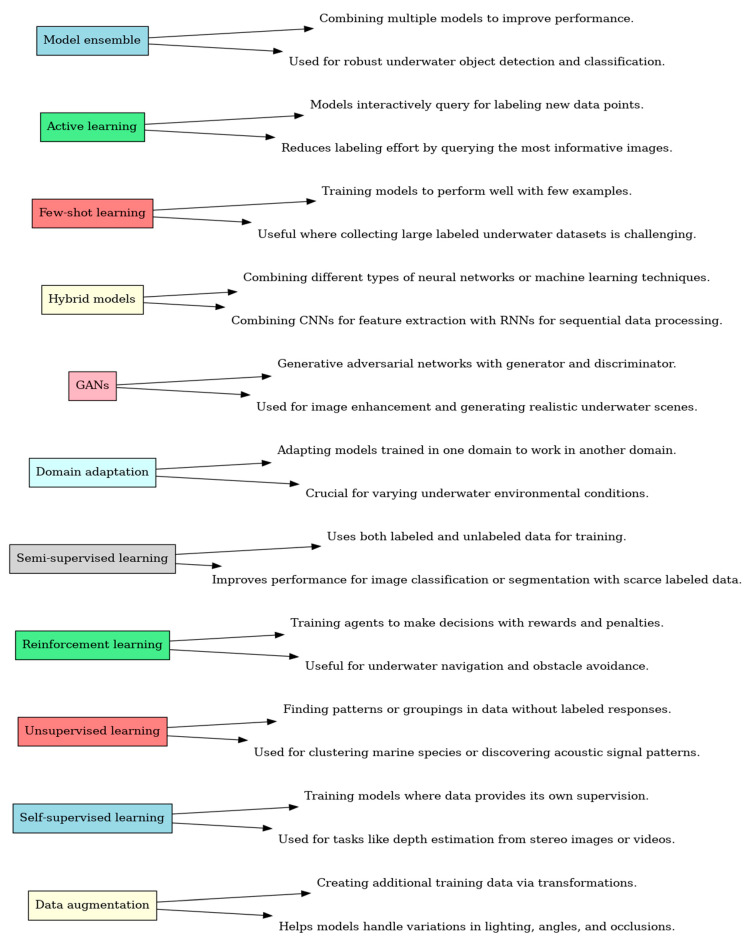
Exploring deep learning techniques for underwater applications, excluding transfer learning.

The graphic in Figure 11 depicts a range of deep learning techniques used in underwater applications. A concise explanation and practical use in underwater settings accompany every method. Techniques such as data augmentation, self-supervised learning [69,118], unsupervised learning [119], reinforcement learning [120,121], semi-supervised learning [122], domain adaptation, generative adversarial networks (GANs), hybrid models, few-shot learning, active learning, and model ensemble are used to improve the effectiveness and efficiency in underwater environments. From here, our next focus is the comparative analysis of underwater SLAM techniques.

## 6. Comparative Analysis of Underwater SLAM Techniques

Underwater simultaneous localization and mapping (SLAM) is essential for unmanned underwater vehicles (UUVs) [92], allowing navigation in the absence of a global positioning system (G.P.S.). While promising, ORB-SLAM3 [123] and DF-VO encounter difficulties facing underwater obstacles, such as limited visibility and light absorption. ORB-SLAM3 [123] performs exceptionally well in low-light conditions but lacks robustness in complicated situations, whereas DF-VO provides superior robustness but requires more extensive processing resources [124,125]. Dark channel prior (D.C.P.), an image processing technique, enhances ORB-SLAM2 [126] by reducing distortions and improving feature matching [127]. Multi-vehicle mapping, utilizing advanced algorithms such as Gaussian mixture robust branch and bound (GMRBnB), surpasses conventional approaches in map registration. Acoustic-based simultaneous localization and mapping (SLAM) utilizes inertial sensors and sonar to accurately navigate conditions with limited vision. This approach incorporates dead reckoning and Bayesian–Gaussian mixtures to create real-time maps [128]. The limitations of VSLAM [129] are addressed by advancements in low-light picture augmentation and self-supervised learning for feature detection [130]. There are still difficulties in accurately aligning maps and dealing with abnormal data points in multi-vehicle SLAM. Integrating various sensors and complex algorithms is crucial for enhancing accuracy and resilience.

Navigation and mapping in underwater environments are difficult; hence, SLAM techniques must be tested. This comparison compares standard and deep learning-based SLAM systems based on accuracy, computing efficiency, resilience, scalability, and sensor fusion. These criteria were chosen because they are crucial to underwater SLAM performance. After a thorough literature analysis, each algorithm was evaluated using RMSE, processing time, and robustness tests. Deep learning-based DeepVO [112] and GAN-SLAM [27] provide superior feature extraction and mapping accuracy, whereas traditional ORB-SLAM [40] and EKF-SLAM are resilient and accurate in underwater applications. The tables below show that deep learning algorithms are better at feature extraction and posture estimation than standard methods, making them appropriate for precise underwater mapping. This comprehensive comparison shows the present status of SLAM technologies and highlights topics for future research, emphasizing the need for resilient, efficient, and accurate underwater SLAM systems.

The above sections provide a qualitative summary of SLAM techniques and their improvements, but a quantitative comparison is needed to reach a complete understanding. Following are the criteria used to compare SLAM methods in each table:

Table 1 compares underwater-applicable classical SLAM algorithms [36,40,44,48,130] based on accuracy, computational efficiency, resilience, scalability, and sensor fusion.

Table 2 compares underwater-applicable deep learning-based SLAM algorithms based on feature extraction, temporal modeling, data augmentation, pose prediction, and mapping accuracy [131,132].

After this clear comparison, the next interest of our dissertation is to elaborate on the advantages and superiority of deep learning relative to conventional methods.

## 7. Advantage and Superiority of Deep Learning Relative to the Conventional Method

**Figure 12:**
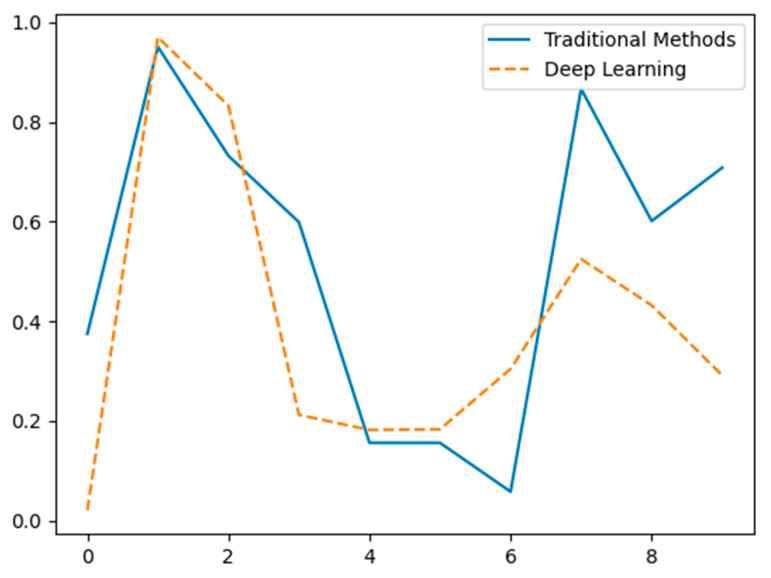
Comparison of feature extraction capabilities between traditional methods and deep learning (hypothetical data).

Figure 12 illustrates the differences in feature extraction performance between traditional methods (solid blue line) and deep learning methods [133,134] (dashed orange line) using hypothetical data. The X-axis represents different data points or feature indices, while the Y-axis indicates the normalized value or significance of the extracted features. Traditional methods show significant variability with pronounced peaks and troughs, suggesting inconsistent feature extraction performance. In contrast, deep learning methods exhibit a more balanced and consistent extraction pattern, highlighting their potential advantage in capturing a broader range of features effectively [135]. The data is hypothetical and intended for conceptual demonstration.

**Figure 13:**
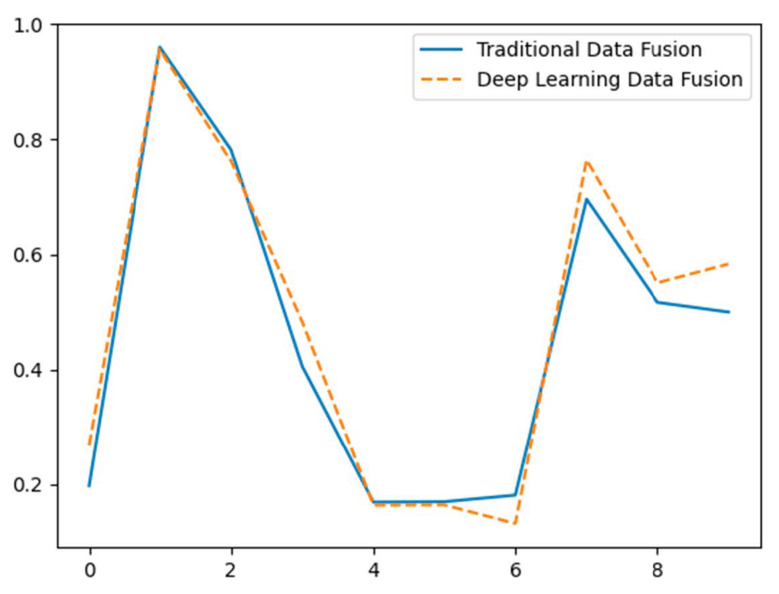
Comparative evaluation of data fusion performance: traditional methods vs. deep learning (hypothetical data).

Figure 13 depicts the disparities in the effectiveness of data fusion between conventional techniques (represented by a solid blue line) and deep learning techniques [99,136,137,138,139,140] (represented by a dashed orange line) using hypothetical data. The X-axis depicts distinct data points, while the Y-axis indicates the standardized value of the combined data from two sensors. Conventional techniques employ a basic mean calculation for data fusion, leading to a direct but maybe less precise amalgamation of sensor data. Deep learning [141,142] approaches utilize a weighted average, showcasing a more complex and presumably more efficient strategy for combining sensor data. The data is fictitious and provided for conceptual purposes.

**Figure 14:**
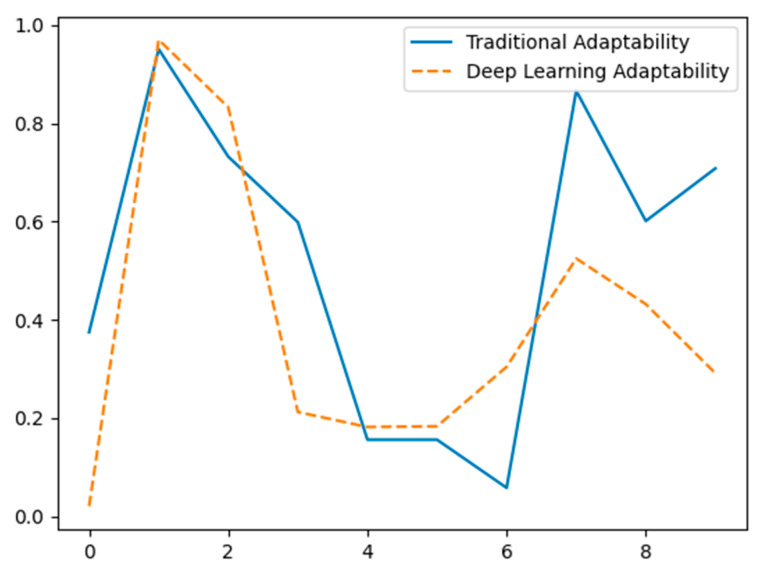
Adaptation to new environments comparison of traditional methods vs. deep learning (hypothetical data).

Figure 14 contrasts the adaptation of classical approaches (solid blue line) versus deep learning methods (dashed orange line) to new contexts using hypothetical data. The X-axis depicts various data points or environmental variables, while the Y-axis shows the normalized adaptation performance statistic. Traditional approaches exhibit heterogeneity in adaptation, indicating possible difficulties in changing to new environments without retraining. On the other hand, deep learning approaches show greater consistency and adaptability, emphasizing their ability to learn and respond more efficiently to changing settings. The data is fictitious and designed for conceptual presentation.

Figure 15 compares the real-time processing performance between classical approaches (represented by a solid blue line) and deep learning methods (represented by a dashed orange line) using hypothetical data. The X-axis shows discrete time intervals or occurrences, whereas the Y-axis indicates the standardized processing durations. Conventional techniques demonstrate longer processing times, indicating a slower performance. On the other hand, deep learning techniques have considerably shorter processing durations, suggesting their capacity to handle data more effectively in real time. The data provided is fictitious and is intended solely for conceptual demonstration purposes.

Figure 16 illustrates the comparative capacities of classical approaches (represented by a solid blue line) and deep learning methods [143,144] (represented by a dashed orange line). The data used in this comparison is hypothetical. The X-axis depicts varying degrees of scene complexity, while the Y-axis indicates the standardized performance in comprehending and analyzing the situations. Conventional approaches exhibit inconsistent results, frequently encountering difficulties when dealing with more complicated scenes. Deep learning [112,114,145] approaches, on the other hand, provide exceptional performance [146], especially in intricate environments, emphasizing their capacity to capture and comprehend advanced semantic information efficiently. The data provided is fictitious and is intended solely for conceptual demonstration purposes.

The hypothetical data results reveal various advantages of deep learning over previous approaches in underwater SLAM navigation. First, deep learning approaches extract features more reliably and precisely than classical methods, which have abrupt peaks and troughs. Understanding underwater settings requires reliable feature extraction. Additionally, deep learning algorithms are more noise-resistant. Traditional approaches [147] are more noise-sensitive, causing more significant feature value variations. In contrast, deep learning approaches, as shown in Figure 14, Figure 15 and Figure 16, preserve more stable and consistent values in noise, improving performance in noisy underwater settings. While straightforward, traditional data fusion approaches employ averaging, which can be inaccurate. Deep learning approaches use weighted averaging to integrate sensor data sources and improve SLAM system accuracy and reliability.

Deep learning also excels at adaptability to hypothetical data, as illustrated in Figure 14, Figure 15 and Figure 16 [148]. Deep learning algorithms [131] adapt to new surroundings more reliably than traditional methods. Due to its higher learning capabilities, deep learning is ideal for dynamic underwater situations. Deep learning algorithms outperform standard methods in complex scenarios for high-level scene understanding. Deep learning algorithms improve interpretation and decision-making in complicated scenes, while traditional methods do poorly in such cases. 

Another benefit of deep learning is real-time processing. Traditional methods take longer to process, indicating slower performance. Deep learning algorithms [20] have far reduced processing times, indicating more efficient real-time processing, which is crucial for underwater SLAM applications. Hypothetical data illustrates this in Figure 14, Figure 15 and Figure 16. 

Finally, traditional methods vary in retaining excellent perception and mapping quality. However, deep learning algorithms provide more accurate and detailed underwater maps across perceptual quality levels.

Therefore, deep learning in underwater SLAM navigation could increase autonomous underwater vehicle accuracy, reliability, and efficiency. Traditional methods have drawbacks, but deep learning uses complicated models and data-driven approaches to overcome them. These algorithms can learn from big datasets, adapt to changing conditions, and integrate numerous sensor data sources better, making them ideal for underwater SLAM navigation. Further research should address constraints like computing requirements and vast training datasets to fully realize these strategies’ benefits in real-world applications.

Deep learning brings several critical advantages to underwater SLAM (simultaneous localization and mapping) navigation, addressing many limitations of traditional methods.

Firstly, feature extraction is a significant benefit, as deep learning models excel at extracting complex and high-level features from raw sensor data [58]. This is particularly useful in underwater environments, where traditional methods often struggle with noise, low visibility, and the lack of distinctive features. Deep learning allows the extraction of more informative and reliable features, even in challenging conditions.

Secondly, deep learning [136,137] algorithms show robustness to noise. Deep learning models can maintain accurate positioning and mapping in underwater settings, where environmental noise and uncertainties often affect sensors. This results in improved SLAM accuracy and system reliability in situations where traditional methods would falter.

Another critical advantage is data fusion. Deep learning [149,150] models can integrate data from multiple types of sensors, such as sonar, LiDAR, and cameras. This ability enhances the SLAM system’s overall performance by providing more robust positioning and mapping, even in cases where individual sensors may give incomplete or erroneous data.

In addition, deep learning [136,137,138,139] enables adaptability and learning. Unlike traditional SLAM approaches that rely on predefined models and assumptions about the environment, deep learning models can learn from the data and adapt to new environments over time. This makes them more flexible and scalable in dynamic underwater conditions where the environment can change unpredictably.

Moreover, deep learning models excel at handling complex dynamics. Underwater environments are highly dynamic and nonlinear, often making it difficult for traditional methods to cope. Deep learning models, with their ability to model complex patterns, improve the accuracy of trajectory estimation and mapping in such settings. Because of these operational advantages, deep learning is superior in understanding complex underwater scenes.

For example, deep learning models such as CNNs (convolutional neural networks) can highly understand scenes. This allows them to interpret and analyze semantic information from underwater scenes that may be too complex for traditional methods, leading to more accurate scene interpretation.

Deep learning [69] also enables end-to-end learning, where raw sensor inputs are processed directly into SLAM outputs without needing handcrafted feature extraction. This reduces complexity in the system design and allows for a more seamless, automated approach to SLAM in underwater settings.

Additionally, deep learning models demonstrate a high level of generalization across environments. Models trained on large and diverse datasets are more likely to adapt to new environments with minimal tuning than traditional methods requiring extensive adjustments to function effectively [151] in different underwater terrains.

Another key benefit is the capability for real-time processing. Deep learning algorithms can process complex underwater scenes in real time, making them suitable for dynamic and time-sensitive SLAM applications, such as autonomous underwater vehicle (AUV) navigation.

Finally, deep learning enhances perception and mapping accuracy. By using deep learning algorithms, SLAM systems can produce more detailed and accurate underwater maps improving perception and navigation [152].

## 8. Conclusions/Significance

While our work summarizes the advances made possible by deep learning in underwater SLAM, several issues still need to be further explored. Deep learning methods have a limited real-time application in dynamic underwater environments because of their high computational resource requirements, even though they are resilient in feature extraction and noise treatment. The requirement for extensive and varied datasets for training presents another difficulty because gathering such data in underwater environments is expensive and challenging. Even with sophisticated algorithms, environmental disturbances, including shifting circumstances, visibility problems, and sensor noise, still impact performance. Furthermore, unmanned underwater vehicles (UUVs) present particular concerns about the high energy consumption linked to operating complicated deep learning models, as energy economy is crucial for prolonged missions. Finally, despite the significant advances in mapping precision and flexibility that deep learning provides, hardware constraints still limit real-time processing. These difficulties show that to fully realize the potential of deep learning in underwater SLAM applications, more research is required in model optimization, effective data gathering, improving data availability through simulations, and exploring hybrid approaches combining deep learning with traditional underwater systems and hardware solutions.

This paper lays a solid groundwork for the progress of unmanned underwater vehicle (UUV) navigation, with a specific emphasis on enhancing AI-SLAM algorithms, specifically those powered by deep learning. Future research will focus on improving various sensor fusion approaches and integrating advanced technologies such as multibeam sonar, stereo cameras, LiDAR, and Imu, as well as methodologies like SBL/USBL [153,154] and DLV [155]. The goal should be to enhance the accuracy of UUV navigation in challenging underwater situations.

Investigating the incorporation of cutting-edge technology like machine learning and enhanced computer vision shows potential for improving the reliability of UUV navigation systems. Ensuring the capacity to adapt to various unmanned underwater vehicles (UUVs) and mission needs is critical. It is imperative to have cooperation between researchers, industry professionals, and policymakers to establish standards and effectively apply these improvements in real-world scenarios.

This work has thoroughly examined underwater simultaneous localization and mapping (SLAM) technologies, specifically focusing on incorporating deep learning methods. We have emphasized their crucial function in improving underwater navigation and perception by analyzing different sensors and their respective uses, such as vision sensors, sonar, and LiDAR. The assessment of various simultaneous localization and mapping (SLAM) algorithms highlighted the progress and difficulties in the field, namely the advantages of integrating deep learning techniques such as convolutional neural networks (CNNs), long short-term memory (LSTM) networks, generative adversarial networks (GANs) and other deep learning methods.

We have shown that the utilization of deep learning greatly enhances the process of extracting features, estimating poses, and fusing data, resulting in underwater SLAM systems that are more precise and resilient. Nevertheless, these systems have difficulties, such as intense computational requirements and the opaque nature of neural networks, which can affect the capacity to interpret and apply them in real time.

## Figures and Tables

**Table 1:** A comparison of traditional underwater SLAM techniques caption.

Algorithm	Accuracy	Computational Efficiency	Robustness	Scalability	Sensor Fusion Capability
ORB-SLAM	High	Moderate	Moderate	High	Moderate
ROVIO	High	High	Moderate	High	Moderate
LSD-SLAM	Moderate	High	Moderate	High	Moderate
DVO-SLAM	High	Moderate	High	Moderate	Low
MSCKF	High	Very high	High	High	High
VSO	Moderate	Very high	Low	Low	Low
VSLAM	High	Moderate	High	High	High
RTAB-Map	Moderate	High	Moderate	High	Low
EKF-SLAM	High	Moderate	High	High	High
Graph-Based SLAM	High	Moderate	High	High	High
GMapping	Moderate	High	Moderate	Moderate	Moderate

**Table 2:** Comparative analysis of deep learning-based underwater SLAM techniques.

Algorithm	Feature Extraction Quality	Temporal Modeling Accuracy	Data Augmentation Efficiency	Pose Estimation Precision	Mapping Accuracy
DeepVO	High	High	Moderate	High	High
PoseNet	High	High	Low	High	Moderate
DVL-SLAM	Moderate	Moderate	Low	Moderate	Moderate
SuperPoint	High	N/A	N/A	High	High
DeepMatcher	High	N/A	N/A	High	High
GAN-SLAM	High	N/A	High	High	High
VPF SLAM	Moderate	N/A	High	Moderate	High
RTAB-Map	High	High	Moderate	High	High
D.S.O.	High	High	Moderate	High	High
ORB-SLAM2	High	High	High	High	High
LOAM	High	High	Moderate	High	High
